# The Separation of Cannabinoids on Sub-2 µm Immobilized Polysaccharide Chiral Stationary Phases

**DOI:** 10.3390/ph14121250

**Published:** 2021-11-30

**Authors:** Takafumi Onishi, Weston J. Umstead

**Affiliations:** 1 Daicel Corporation, CPI Company, 1-1 Shinko-cho, Myoko-shi 944-8550, Niigata, Japan; tk_onishi@jp.daicel.com; 2 Chiral Technologies, Inc., 1475 Dunwoody Drive, Suite 310, West Chester, PA 19380, USA

**Keywords:** cannabinoids, UHPLC, polysaccharide chiral stationary phases, normal phase, reversed phase, chiral chromatography

## Abstract

The increased use and applicability of Cannabis and Cannabis-derived products has skyrocketed over the last 5 years. With more and more governing bodies moving toward medical and recreational legalization, the need for robust and reliable analytical testing methods is also growing. While many stationary phases and methods have been developed for this sort of analysis, chiral stationary phases (CSPs) are unique in this area; not only can they serve their traditional chiral separation role, but they can also be used to perform achiral separations. Given that mixtures of cannabinoids routinely contain enantiomers, diastereomers, and structural isomers, this offers an advantage over the strictly achiral-only analyses. This work presents the separation of a 10-cannabinoid mixture on several polysaccharide-based sub-2 µm CSPs with both normal-phase and reversed-phase ultra-high-performance liquid chromatography (UHPLC) conditions. Along with the separation of the mixture, appropriate single-peak identification was performed to determine the elution order and reported where applicable.

## 1. Introduction

Like many other naturally sourced materials, Cannabis contains a complex blend of identified and unidentified compounds ranging from major and minor cannabinoids, terpenes, derivatives of these compounds, and other commonly occurring plant-based compounds such as chlorophyll [1,2,3]. This can make the accurate analysis of Cannabis for the levels of tetrahydrocannabinol (THC), levels of other cannabinoids, and presence of pesticides (as examples) challenging and complicated.

With the passage of the Controlled Substances Act of 1970 in the United States of America, which classified marijuana as a Schedule I drug, the need for any quality control or purity analysis of Cannabis-related products became moot due to its legal status. More recently, however, with the passage of the 2018 Farm Bill in the USA, hemp-based products were legalized with the strict regulation that they must contain less than 0.3% by weight of THC. Since then, efforts have been refocused on the development of applications to address the accurate quantification and removal of THC [4,5]. Additionally, applications have also been present for the analysis and quantification of other major and minor cannabinoids, pesticide testing, and terpene/terpenoid analysis [6,7,8,9,10,11,12,13,14,15,16,17].

High-performance liquid chromatography (HPLC) or some variation of super-critical fluid chromatography (SFC) or extraction (SFE) have been the go-to technologies up to this point for such applications. As the field is well established, offering a wide variety of chiral and achiral stationary phases, a laboratory or producer is presented with many flexible options to choose from, depending on their set-up, budget limitations, and hands-on experience.

Despite numerous publications on the topic, including several works on polysaccharide chiral columns [18,19,20], there have been no reports to date utilizing sub-2 µm polysaccharide-based chiral stationary phases (CSPs). While most cannabinoids do not naturally occur as a pair of enantiomers, for instance, cannabidiol (CBD) or cannabigerol (CBG), there are a few pairs that do, such as Δ^8^ and Δ^9^ THC. Moreover, while one enantiomer might not be naturally occurring, with new synthetic routes emerging for production and consumption, these non-naturally occurring enantiomers are emerging, and the need to quantify them is important.

**Figure 1:**
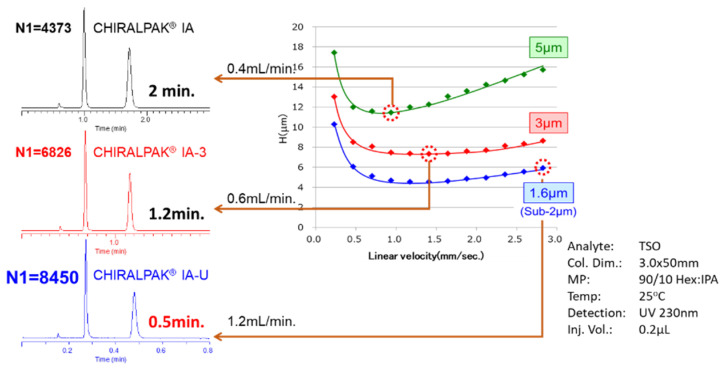
Van Deemter plot for varying particle sizes of Chiralpak IA immobilized polysaccharide CSP.

Figure 1, which was generated by the authors using the conditions shown below, displays an overlay of three Van Deemter plots showing the performance of 5 µm, 3 µm, and sub-2 µm immobilized amylose tris [3,5-dimethylphenyl] carbamate CSPs. The y axis is a representation of H or the theoretical plate height (in µm), and the x axis is linear velocity (in mm/s). Due to the larger particle size, the 5 and 3 µm CSPs (in green and red respectively) have a naturally higher theoretical plate-height, due to the nature of packing a column with a larger particle. This translates into a lower theoretical plate-count and a lower resolution. For both particle sizes, as the linear velocity is increased, the theoretical plate-height increases, which will equate to a further loss of resolution. The smaller sub-2 µm particle size is more efficient to pack, which translates into a smaller theoretical plate-height. As the linear velocity increases, the loss of resolution is less noticeable. This translates into sub-2 µm CSPs being capable of an increased separation capacity, higher theoretical plate-count, and faster analysis times without a loss of separation. Therefore, for complex mixtures such as those coming from Cannabis, it would stand to reason they would be well suited for the separation of enantiomers, diastereomers, structural isomers, and other isomers, all in one method. The only caveat is that a UHPLC, or ultra-high performance liquid chromatography instrument, should be used when implementing sub-2 µm columns, as these instruments are optimized with minimal extra-column volume and high-pressure pump capabilities to take advantage of these benefits.

This work presents new applications for the separation of a 10-cannabinoid mixture under both normal-phase and reversed-phase UHPLC conditions, with several Daicel sub-2 µm CSPs. When available, individual standards were also run to demonstrate the elution order of the cannabinoids under the indicated methods. In some cases, two columns were coupled to improve the separation of closely eluting compounds. The fastest, best, or both analysis conditions are presented.

## 2. Results

### 2.1. Normal Phase

The screening of the 10-cannabinoid mixture was performed under normal-phase conditions of 90-10-0.1 = Hex-EtOH-TFA and 90-10-0.1 = Hex-IPA-TFA. This was chosen based on a retention check of the mixture, which demonstrated that analytes were not eluting too quickly from the column, but also that there was sufficient retention to afford a separation. All sub-2 µm immobilized polysaccharide columns described in the Materials and Methods section were screened with these conditions, with baseline separations observed on nearly all columns. For the purpose of achieving a baseline resolution for all cannabinoids in the mixture, which is covered in the Discussion section, the separations on Chiralpak IB-U and Chiralpak IH-U showed the best initial separation and the most promise for complete baseline resolution. The conditions which yielded baseline separation of all cannabinoids are presented in Table 1. Additional conditions are provided in the Supplementary Materials for faster analyses.

### 2.2. Reversed Phase

Similar to the normal-phase screening, the 10-cannabinoid mixture was screened on all sub-2 µm immobilized polysaccharide columns to start, utilizing a gradient of water/acetonitrile or water/methanol, with 0.1% TFA in the aqueous as an additive. The gradient started at 80% water and decreased to 10% water (or increased from 20% organic to 90% organic) over a 12 min period. For ease of use, during optimization, the gradients were converted to isocratic methods, with acetonitrile being the preferred organic.

Methanol, although it yielded separation, resulted in extremely long retention, and was therefore not as useful as Acetonitrile, which provided moderate retention and selectivity. Again, as with the normal-phase screening, all columns showed separations of some or nearly all cannabinoids. For the purpose of achieving a baseline resolution for all cannabinoids, Chiralpak ID-U independently, or coupled with IC-U and IG-U, showed the most promise for complete baseline resolution. The conditions which yielded baseline separation of all cannabinoids are presented in Table 2. Additional conditions are provided in the Supplementary Materials for faster analyses.

### 2.3. A Note on Optimization

The purpose of this work, as stated in the introduction, is to share new applications for the separation of complex cannabinoid mixtures, and to attempt to achieve baseline resolution for all components. This means the methods presented are not specifically optimized, other than to attempt to achieve baseline separation. Ultimately, optimization is intended for the end-user of these applications, where the focus can be on a specific set or sets of cannabinoids. This is most prominently demonstrated in the reversed-phase applications, where the very long retention of THCA-A could be resolved by using a gradient. Furthermore, better resolution between earlier, more-closely eluting cannabinoids could be increased by decreasing the strength of the mobile phase or decreasing the flow rate. Due to this, no specific separation parameters, such as resolution, selectivity, or k’ values, are shared in this work for these applications.

## 3. Discussion

The separations under normal-phase conditions were focused on two applications: the best separation (baseline resolution of all components) and the fastest analysis. It is important to note that not all chromatograms are shared within the body of this text, but are labeled and can be found in the Supplementary Materials.

**Figure 2:**
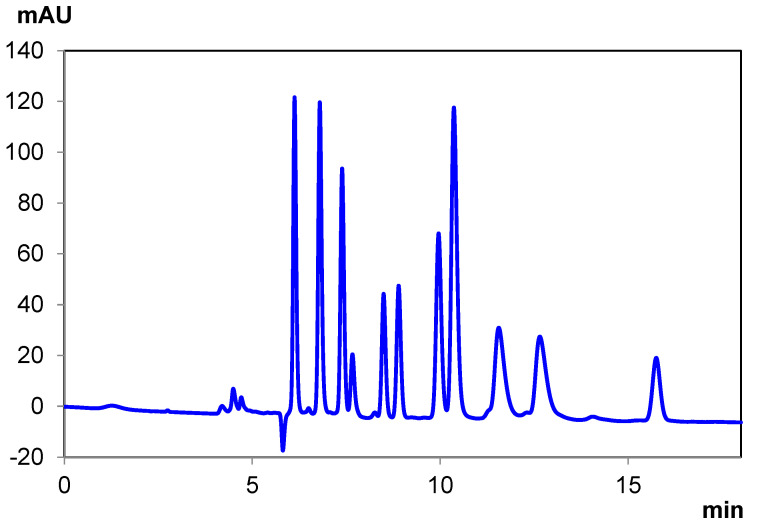
Ten-cannabinoid mixture separation under normal-phase conditions with Chiralpak IB-U.

Generally, the resolving power of a single sub-2 µm column was not sufficient to achieve baseline resolution in all cases; therefore, it was often required to couple two columns together. This is the case for the separations depicted in Figure 2 and Figure S1 (Supplementary Materials) on IB-U. Some alcohol is required to ensure timely elution of several of the cannabinoids. However, too much alcohol, particularly Ethanol, yields coelution of several earlier eluting cannabinoids.

It is for this reason that a mixture of Isopropanol and Ethanol was utilized, as this yields a compromise between retention and timely elution. However, this still resulted in some coelution, namely peaks 3/4 and 5/6.

This can be improved by reducing the flow rate from 0.425 mL/min to 0.21 mL/min and decreasing the elution strength of the mobile phase by increasing the percentage of hexane by 0.3%. This change resolved the coelution between peaks 3/4 and 5/6.

Analysis of individual cannabinoid standards demonstrated the elution order of the cannabinoid mixture on IB-U (Figure S2, Supplementary Materials) as follows, starting with: Tetrahydrocannabinolic Acid A (THCA-A), Cannabidiolic Acid (CBDA), delta-8 Tetrahydrocannabinol (Δ^8^-THC), Cannabidiol (CBD), (±)-Cannabichromene (CBC), Cannabinol (CBN), delta-9 Tetrahydrocannabinol (Δ^9^-THC), and Cannabigerol (CBG) (from first to last—a full description of cannabinoids and abbreviations is available in the Methods and Materials section). All elution times are also explicitly listed in Table 3. As it happens, peaks 3 and 4 are a critical pair of cannabinoids, delta-8 Tetrahydrocannabinol (Δ^8^ THC) and Cannabidiol (CBD). While perhaps not as critical, peaks 5 and 6 are the enantiomers of Cannabichromene (CBC), which cannot be resolved on standard achiral stationary phases.

**Figure 3:**
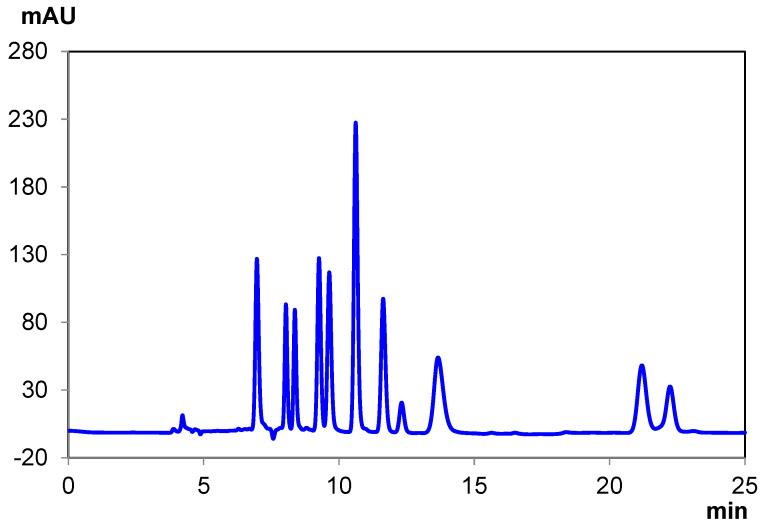
Ten-cannabinoid mixture separation under normal-phase conditions with Chiralpak IH-U.

The separations on Chiralpak IH-U required a similar approach to achieve baseline resolution on IB-U, in that some alcohol was required but only a minor percentage by volume, and that two columns coupled together provided better resolving power for closely eluting pairs. Figure 3 and Figure S3 (Supplementary Materials) show the best and fastest separations, respectively, with elution times explicitly listed in Table 4. Compared to the separation on IB-U, IH-U shows several changes to the cannabinoid elution order (Figure S4, Supplementary Materials). The specific elution order is: Tetrahydrocannabinolic Acid A (THCA-A), (±)-Cannabichromene (CBC), delta-8 Tetrahydrocannabinol (Δ^8^-THC), delta-9 Tetrahydrocannabinol (Δ^9^-THC), Cannabinol (CBN), Cannabidiol (CBD), Cannabidiolic Acid (CBDA), and Cannabigerol (CBG) (from first to last). Of note are the earlier elution of delta-9 Tetrahydrocannabinol (Δ^9^ THC) and a later elution of CBD. For applications wherein the accurate quantification of the removal of THC from CBD products is required, the analysis on IH-U would likely be better suited given the higher degree of separation of these components.

Reversal of the elution order is not entirely uncommon for chiral separations when switching between different chiral stationary-phase and mobile-phase combinations ([21,22] as examples). There have been numerous studies aimed at making a connection between the polysaccharide backbone, chiral selector substitution, and electronics, as well as analytes [23,24,25]. What is known with certainty is that there are a number of complicated and usually unpredictable intermolecular interactions which take place between the chiral stationary phase and the analyte. These include, but are not limited to, hydrogen bonding, π–π stacking, Van der Waals forces, and dipole–dipole interactions. These combinations, with the additional variable of mobile-phase composition, make predicting the retention, elution order, and selectivity very challenging. This is most certainly the case for the separation of cannabinoid mixtures as well. These on-column interactions are, at least partly, the explanation for the observed elution-order changes.

**Figure 4:**
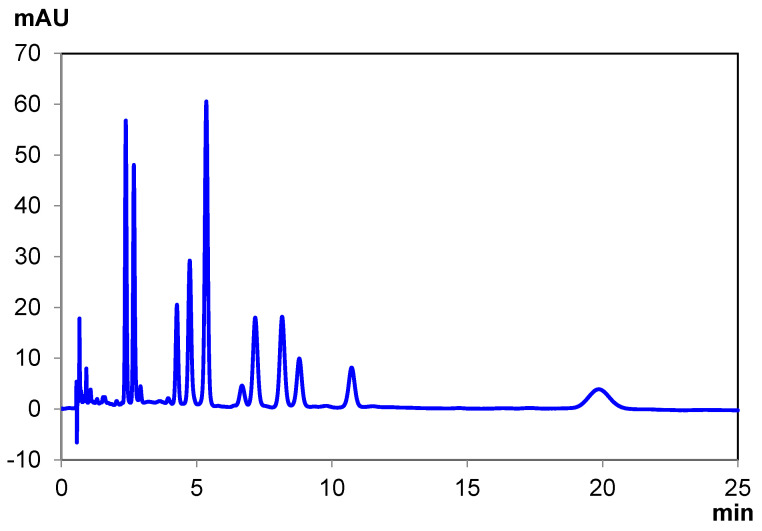
Ten-cannabinoid mixture separation under reversed-phase conditions with Chiralpak IG-U.

The reversed-phase separations took the same approach as the normal-phase separations, with a focus on the best separation and the fastest separation. Chiralpak IG-U produced a baseline resolution for all cannabinoids in the mixture, without the need for coupled columns (as shown in Figure 4). As described in the results section, the initial gradient method for the screening was converted to an isocratic method. Polysaccharide columns can be used with gradient methods, but isocratic methods do not require the subsequent column re-equilibration of a gradient method; therefore, isocratic methods could be preferred when repeated, faster analysis is required. Unlike with the normal-phase separations, the reversed-phase separation conditions require a considerably higher percentage of polar organic solvent to elute the cannabinoids. At 55% by volume acetonitrile, the retention is still rather high, so an increased flow rate of 0.80 mL/min can be used, given the lower pressure of only using one column.

Not surprisingly, changes in the elution order are again observed when compared to the normal-phase separations (Figure S5, see references [21,22,23,24,25]), with elution times explicitly listed in Table 5. Specifically, the elution was: CBDA, CBG, CBN, CBD, delta-8 THC, delta-9 THC, CBC, and THCA-A (first to last). One might note that the latter eluting cannabinoids from the normal-phase separations tend to elute much quicker under reversed-phase conditions, and vice versa for the earlier eluting cannabinoids. The chiral stationary phase was different for the normal-phase separations compared to reversed-phase separations and could play a role in this. However, even when comparing IB-U to IH-U, there was not as dramatic a shift in the elution order compared to the reversed-phase methods. A change in mobile phase, particularly the water and acetonitrile, more likely promotes a stronger interaction between the analyte and CSP, causing this shift. Of particular note is THCA-A, which elutes first under normal-phase conditions and is retained by more than 20 min longer under reversed-phase conditions.

**Figure 5:**
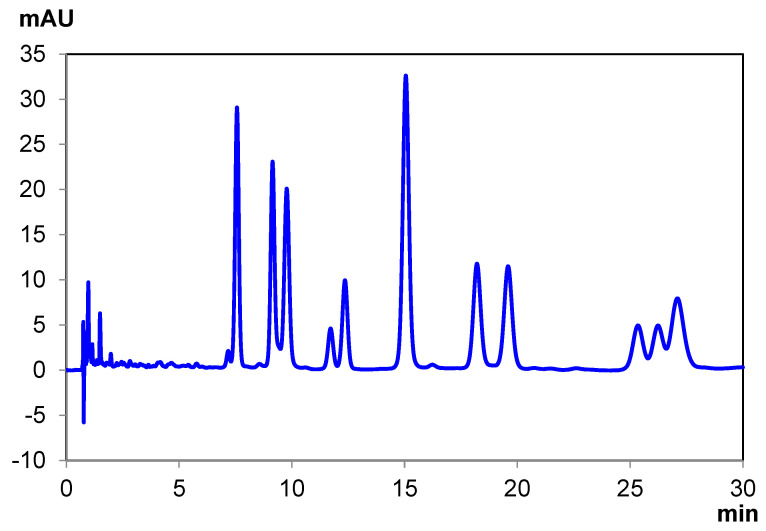
Ten-cannabinoid mixture separation under reversed-phase conditions with Chiralpak ID-U.

Chiralpak ID-U produces a similarly efficient separation of the 10-cannabinoid mixture under reversed-phase conditions (Figure 5). To better resolve several of the closer-eluting cannabinoids, the flow rate was reduced to 0.6 mL/min, and the cannabinoids were retained for longer; the latter was achieved by decreasing the strength of the mobile phase from 45% aqueous in the separation on IG-U to 55% aqueous on ID-U. However, as can be seen, there are still several coelutions, particularly of the three later-eluting cannabinoids. The elution order and times are explicitly listed in Table 6.

**Figure 6:**
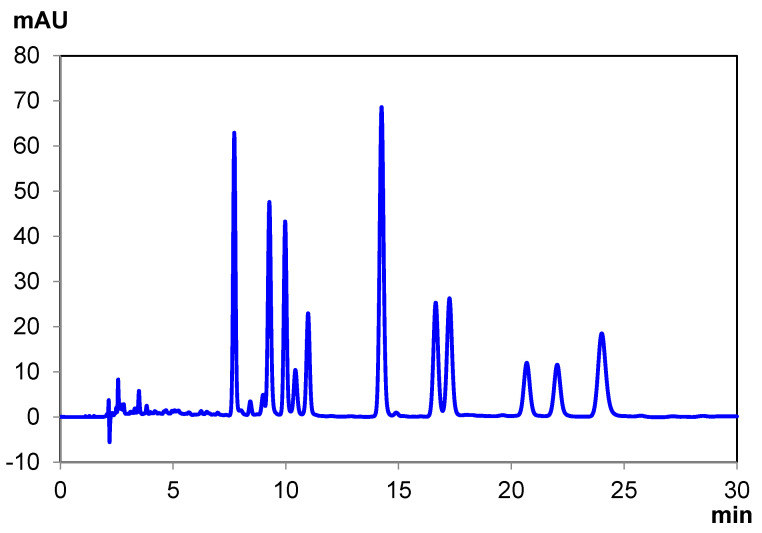
Ten-cannabinoid mixture separation under reversed-phase conditions with Chiralpak ID-U+IC-U.

This can be resolved by coupling ID-U with IC-U. With the higher pressure coming from two coupled columns, the flow rate needed to be reduced to 0.425 mL/min; however, the elution strength of the mobile phase can be increased to counter-balance this. The coupling and condition modifications resolve these previously observed coelutions, resulting in a baseline resolution for all cannabinoids in the mixture (Figure 6). A check of the elution order (Figure S6, Supplementary Materials) shows that several of the cannabinoids have shifted slightly, which could make this a more amenable analysis, depending on the desired application. The elution times are explicitly listed in Table 7. It is also important to note that the coupling does not change any elution order from the ID-U separation alone (Figure S7, Supplementary Materials) but simply improves the resolution.

## 4. Materials and Methods

**Figure 7:**
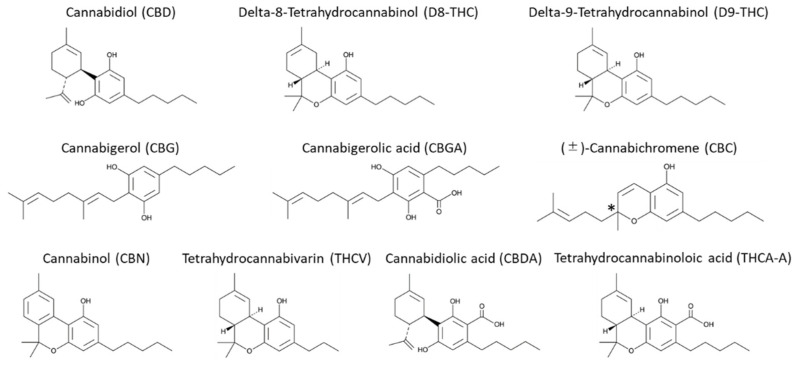
Structures of cannabinoids in 10-phytocannabinoid mixture.

The 10-cannabinoid mixture and individual cannabinoid standards were purchased from Cayman Chemical Company in Ann Arbor, MI, USA as 1.0 mg/mL ampules, and diluted individually as indicated in the figures for each analysis. The 10-cannabinoid mixture contained Tetrahydrocannabivarin (THCV), Cannabidiolic Acid (CBDA), Cannabigerolic Acid (CBGA), Cannabigerol (CBG), Cannabidiol (CBD), Cannabinol (CBN), Tetrahydrocannabinolic Acid A (THCA-A), delta-9 Tetrahydrocannabinol (Δ^9^-THC), delta-8 Tetrahydrocannabinol (Δ^8^-THC), and (±)-Cannabichromene (CBC). The structures of all cannabinoids are shown in Figure 7. The 10-cannabinoid mixture was screened on Daicel’s immobilized sub-2 µm chiral column series, which included Chiralpak IA-U [amylose tris (3,5-dimethylphenylcarbamate)], IB-U [cellulose tris (3,5-dimethylphenylcarbamate)], IC-U [cellulose tris (3,5-dichlorophenylcarbamate)], ID-U [amylose tris (3-chlorophenylcarbamate)], IG-U [amylose tris (3-chloro-5-methylphenylcarbamate)], and IH-U [amylose tris (S)-α-methylbenzylcarbamate. All columns were of dimensions 3.0 mm i.d. × 100 mm L.

All screening and method optimization was performed on an Agilent 1290 Infinity II UHPLC equipped with a 1290 Infinity II Flexible Pump and a 1290 Infinity II Diode Array Detector. All solvents were HPLC grade or higher and were purchased from the Scientific Equipment Company (Aston, PA, USA). Specifically, the hexanes used contained 95% n-hexane, and the Ethanol was Reagent Alcohol (90% Ethanol with 5% Methanol and 5% Isopropanol *v/v/v*). Trifluoroacetic acid was purchased from Sigma-Aldrich (St. Louis, MI, USA) and used as is. Other experimental parameters such as the temperature, flow rate, and injection volume varied across the different analyses to fully optimize the conditions and are indicated in Table 1 and Table 2.

## 5. Conclusions

The accurate quantification of cannabinoids in Cannabis and Cannabis-derived products has seen a significant increase since 2018. Many methods have been published for potency testing, THC content analysis, minor cannabinoid quantification, and more. This work is the first to present such separations on Daicel’s sub-2 µm immobilized polysaccharide chiral stationary phases, both under normal-phase and reversed-phase UHPLC conditions. Although specifically designed for chiral separations, polysaccharide chiral columns can also be used for achiral or structural isomer separations, making them well suited for the analysis of complex cannabinoid mixtures.

When compared to the applications currently available for similar analyses, which are typically on silica or ODS-based achiral columns, these new methods on polysaccharide chiral stationary phases offer a few advantages and improvements. First, and most obvious, is their ability to separate chiral cannabinoids, such as CBC or THC. This cannot be achieved on a standard achiral column. As regulatory oversight increases, being able to separate and quantify these enantiomeric pairs will likely increase as well. Second, while achiral columns are readily available from multiple vendors, and are often initially less expensive, the polysaccharide chiral columns can be used in various mobile-phase modes for both chiral and achiral separations, increasing their utility across multiple application areas.

Under normal-phase conditions, IB-U and IH-U provided two sub-15 min analysis methods, which showed baseline resolution of all or most of the cannabinoids contained within the 10-cannabinoid mixture. Under reversed-phase conditions, ID-U, ID-U+IC-U, and IG-U provided similarly fast analyses with baseline resolution of all cannabinoids. The added ability of the chiral columns to resolve the two enantiomers of CBC, which cannot be achieved on achiral stationary phases, is also demonstrated on all columns. Given the various mobile-phase and column combinations provided, these methods can be adapted to meet a wide range of laboratory needs.

As this work was only performed on clean, analytical standards, the next step for utilizing these methods would be to check the separations on real-world samples. Additionally, the separations could be checked with the addition of other cannabinoids or terpenes to further complicate the analysis. With the added complexity of such samples, further optimization might be required, and can be achieved with the methods provided.

Daicel Corporate Disclaimer: As a responsible provider of quality products and services, Daicel Chiral Technologies provides analytical techniques, which may be of use to a broad range of customers and applications. It does not, however, support or promote the use of its products or services in connection with any contraband activities or products related to Cannabis; this includes, but is not limited to, illegal or illicit drug manufacturing, testing, or consumption.

## Figures and Tables

**Table 1:** Summary of normal-phase analysis conditions.

	Chiralpak IB-U	Chiralpak IH-U
Column Dimension	3.0 mm i.d. × 100 mm L (2 columns coupled)	3.0 mm i.d. × 100 mm L (2 columns coupled)
Mobile Phase	96/3/1/0.1 = n-Hexane/Isopropanol/Ethanol/Trifluoroacetic acid (*v/v/v/v*)	96/3/1/0.1 = n-Hexane/Isopropanol/Ethanol/Trifluoroacetic acid (*v/v/v/v*)
Flow Rate	0.21 mL/min	0.425 mL/min
Temperature	25 °C (controlled)	25 °C (controlled)
Detection	220 nm UV	220 nm UV
Sample	10-cannabinoid mixture (1) 0.1 mg/mL in Hexane/IPA/EtOH = 96/3/1	10-cannabinoid mixture (1) 0.1 mg/mL in Hexane/IPA/EtOH = 96/3/1
Injection Volume	0.5 µL	0.5 µL

**Table 2:** Summary of reversed-phase analysis conditions.

	Chiralpak IG-U	Chiralpak ID-U	Chiralpak ID-U+IC-U
Column Dimension	3.0 mm i.d. × 100 mm L	3.0 mm i.d. × 100 mm L	3.0 mm i.d. × 100 mm L (2 columns coupled)
Mobile Phase	45/55/0.1 = Water/Acetonitrile/Trifluoroacetic acid (*v/v/v*)	55/45/0.1 = Water/Acetonitrile/Trifluoroacetic acid (*v/v/v*)	47.5/52.5/0.1 = Water/Acetonitrile/Trifluoroacetic acid (*v/v/v*)
Flow Rate	0.800 mL/min	0.600 mL/min	0.425 mL/min
Temperature	25 °C (controlled)	25 °C (controlled)	25 °C (controlled)
Detection	220 nm UV	220 nm UV	220 nm UV
Sample	10-cannabinoid mixture (1) 0.1 mg/mL in Water/ACN = 47.5/52.5	10-cannabinoid mixture (1) 0.1 mg/mL in Water/ACN = 47.5/52.5	10-cannabinoid mixture (1) 0.1 mg/mL in Water/ACN = 47.5/52.5
Injection Volume	0.5 µL	0.5 µL	0.5 µL

**Table 3:** Elution times for 10-cannabinoid normal-phase separation on Chiralpak IB-U.

	THCA-A	CBDA	Δ^8^ THC	CBD	CBC	CBN	Δ^9^ THC	CBG	Total Time
Elution Time (min)	6.12	6.80	7.38	7.66	8.49 8.89	10.36	11.56	15.75	18.00

**Table 4:** Elution times for 10-cannabinoid normal-phase separation on Chiralpak IH-U.

	THCA-A	CBC	Δ^8^ THC	Δ^9^ THC	CBN	CBD	CBDA	CBG	Total Time
Elution Time (min)	6.97	8.04 8.37	9.26	9.64	10.62	12.32	13.66	22.23	25.00

**Table 5:** Elution times for 10-cannabinoid reversed-phase separation on Chiralpak IG-U.

	CBDA	CBG	CBN	CBD	Δ^8^ THC	Δ^9^ THC	CBC	THCA-A	Total Time
Elution Time (min)	2.39	4.28	5.37	6.68	7.17	8.16	8.79 10.74	19.81	25.00

**Table 6:** Elution times for 10-cannabinoid reversed-phase separation on Chiralpak ID-U.

	CBDA	CBD	CBG	CBN	Δ^8^ THC	Δ^9^ THC	CBC	THCA-A	Total Time
Elution Time (min)	7.56	11.71	12.35	15.05	18.21	19.59	25.34 26.23	27.10	30.00

**Table 7:** Elution times for 10-cannabinoid reversed-phase separation on Chiralpak ID-U+IC-U.

	CBDA	CBD	CBG	CBN	Δ^8^ THC	Δ^9^ THC	CBC	THCA-A	Total Time
Elution Time (min)	7.71	10.42	10.98	14.24	16.64	17.26	20.88 22.03	24.01	30.00

## Data Availability

Data are contained within the article. The data presented in this study are available in The Separation of Cannabinoids on sub-2 µm Immobilized Polysaccharide Chiral Stationary Phases. Additional data are also available in the Supplementary Materials.

## Supplementary Materials

The following are available online at https://www.mdpi.com/article/10.3390/ph14121250/s1: Table S1: Fastest 10-cannabinoid mixture separation under normal-phase conditions with Chiralpak IB-U—Analysis Conditions; Figure S1: Fastest 10-cannabinoid mixture separation under normal-phase conditions with Chiralpak IB-U; Table S2: Peak Identification for the separation under normal-phase conditions with Chiralpak IB-U—Analysis Conditions; Figure S2: Peak Identification for the separation under normal-phase conditions with Chiralpak IB-U; Table S3: Fastest 10-cannabinoid mixture separation under normal-phase conditions with Chiralpak IH-U—Analysis Conditions; Figure S3: Fastest 10-cannabinoid mixture separation under normal-phase conditions with Chiralpak IH-U; Table S4: Peak Identification for the separation under normal-phase conditions with Chiralpak IH-U—Analysis Conditions; Figure S4: Peak Identification for the separation under normal-phase conditions with Chiralpak IH-U; Table S5: Peak Identification for the separation under reversed-phase conditions with Chiralpak IG-U—Analysis Conditions; Figure S5: Peak Identification for the separation under reversed-phase conditions with Chiralpak IG-U; Table S6: Peak Identification for the separation under reversed-phase conditions with Chiralpak ID-U+IC-U—Analysis Conditions; Figure S6: Peak Identification for the separation under reversed-phase conditions with Chiralpak ID-U + IC-U; Table S7: Peak Identification for the separation under reversed-phase conditions with Chiralpak ID-U—Analysis Conditions; Figure S7: Peak Identification for the separation under reversed-phase conditions with Chiralpak ID-U.
