# Supplementary material for: The Separation of Cannabinoids on Sub-2 µm Immobilized Polysaccharide Chiral Stationary Phases
Source: Pharmaceuticals (Basel). 2021 Nov 30;14(12):1250. doi: 10.3390/ph14121250 (PMC8704058; doi:10.3390/ph14121250)
Supplement: Supplementary file 1 [file pharmaceuticals-14-01250-s001.zip › pharmaceuticals-1392554-supplementary.pdf]

# Supplementary

# The Separation of Cannabinoids on sub-2 μm Immobilized Polysaccharide Chiral Stationary Phases

**Takafumi Onishi [1], Weston J. Umstead [2,*]**

**Table S1**: Fastest ten-cannabinoid mixture separation under normal phase conditions with Chiralpak IB-U – Analysis Conditions

| | **Chiralpak IB-U** |
|---|---|
| Column Dimension | 3.0 mm i.d. × 100 mm L (2 columns coupled) |
| Mobile Phase | 95.7/3.3/1/0.1 = n-Hexane/Isopropanol/Ethanol /Trifluoroacetic acid (v/v/v/v) |
| Flow Rate | 0.425 ml/min |
| Temperature | 25°C (controlled) |
| Detection | 220 nm UV |
| Sample | 10 cannabinoid mixture (1) 0.1 mg/ml in Hexane/IPA/EtOH = 96/3/1 |
| Injection Volume | 0.5 μl |

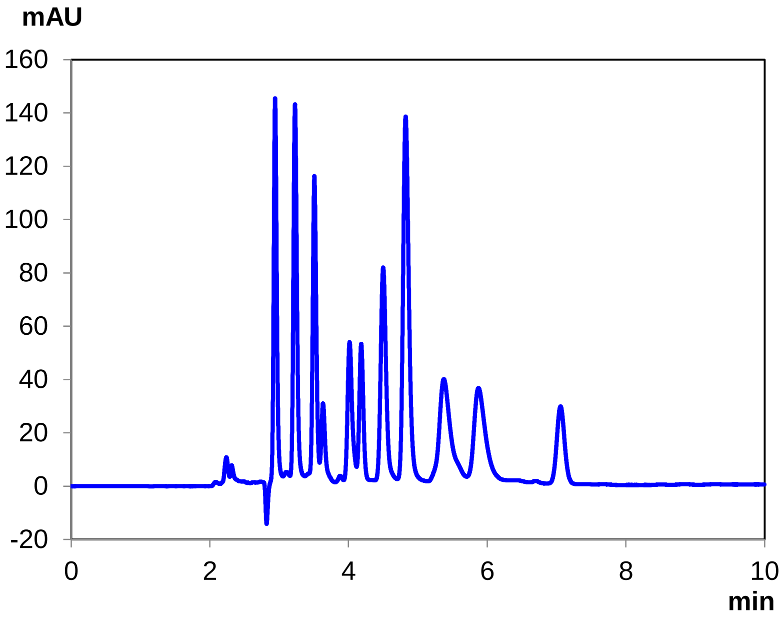


**Figure S1**: Fastest ten-cannabinoid mixture separation under normal phase conditions with Chiralpak IB-U - Chromatogram

**Table S2**: Peak Identification for the separation under normal phase conditions with Chiralpak IB-U – Analysis Conditions

| | **Chiralpak IB-U** |
|---|---|
| Column Dimension | 3.0 mm i.d. × 100 mm L (2 columns coupled) |
| Mobile Phase | 96/3/1/0.1 = n-Hexane/Isopropanol/Ethanol /Trifluoroacetic acid (v/v/v/v) |
| Flow Rate | 0.318 ml/min |
| Temperature | 25°C (controlled) |
| Detection | 220 nm UV |
| Sample | 10 cannabinoid mixture (1) 0.1 mg/ml in Hexane/IPA/EtOH = 96/3/1 Individual Samples: 1.0 mg/ml in Hexane/IPA/EtOH = 96/3/1 |
| Injection Volume | 10 cannabinoid mixture - 0.5 µl Individual Samples – 0.1 µl |

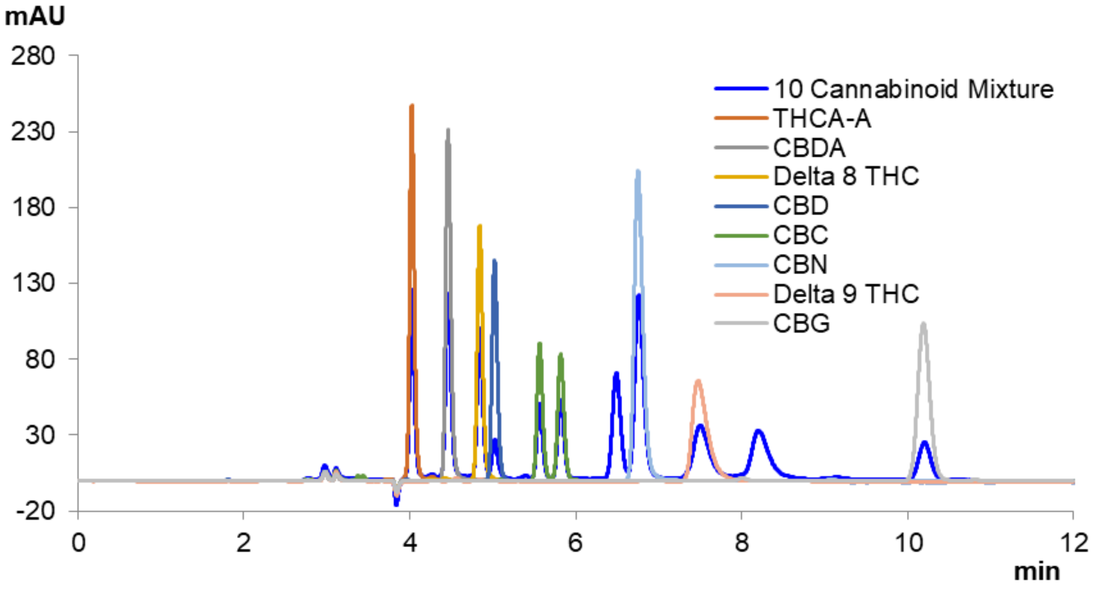


**Figure S2**: Peak Identification for the separation under normal phase conditions with Chiralpak IB-U

**Table S3**: Fastest ten-cannabinoid mixture separation under normal phase conditions with Chiralpak IH-U – Analysis Conditions

| | **Chiralpak IH-U** |
|---|---|
| Column Dimension | 3.0 mm i.d. × 100 mm L (2 columns coupled) |
| Mobile Phase | 96/3/1/0.1 = n-Hexane/Isopropanol/Ethanol /Trifluoroacetic acid (v/v/v/v) |
| Flow Rate | 0.425 ml/min |
| Temperature | 25°C (controlled) |
| Detection | 220 nm UV |
| Sample | 10 cannabinoid mixture (1) 0.1 mg/ml in Hexane/IPA/EtOH = 96/3/1 |
| Injection Volume | 0.5 µl |

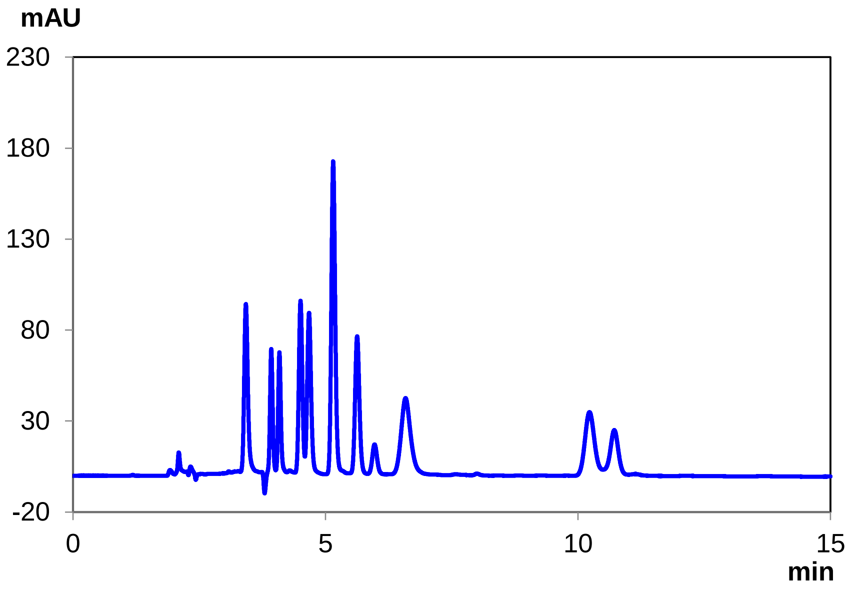


**Figure S3**: Fastest ten-cannabinoid mixture separation under normal phase conditions with Chiralpak IH-U

**Table S4**: Peak Identification for the separation under normal phase conditions with Chiralpak IH-U – Analysis Conditions

| | Chiralpak IH-U |
|---|---|
| Column Dimension | 3.0 mm i.d. × 100 mm L (2 columns coupled) |
| Mobile Phase | 96/3/1/0.1 = n-Hexane/Isopropanol/Ethanol /Trifluoroacetic acid (v/v/v/v) |
| Flow Rate | 0.425 ml/min |
| Temperature | 25°C (controlled) |
| Detection | 220 nm UV |
| Sample | 10 cannabinoid mixture (1) 0.1 mg/ml in Hexane/IPA/EtOH = 96/3/1 Individual Samples: 1.0 mg/ml in Hexane/IPA/EtOH = 96/3/1 |
| Injection Volume | 10 cannabinoid mixture - 0.5 μl Individual Samples – 0.1 μl |

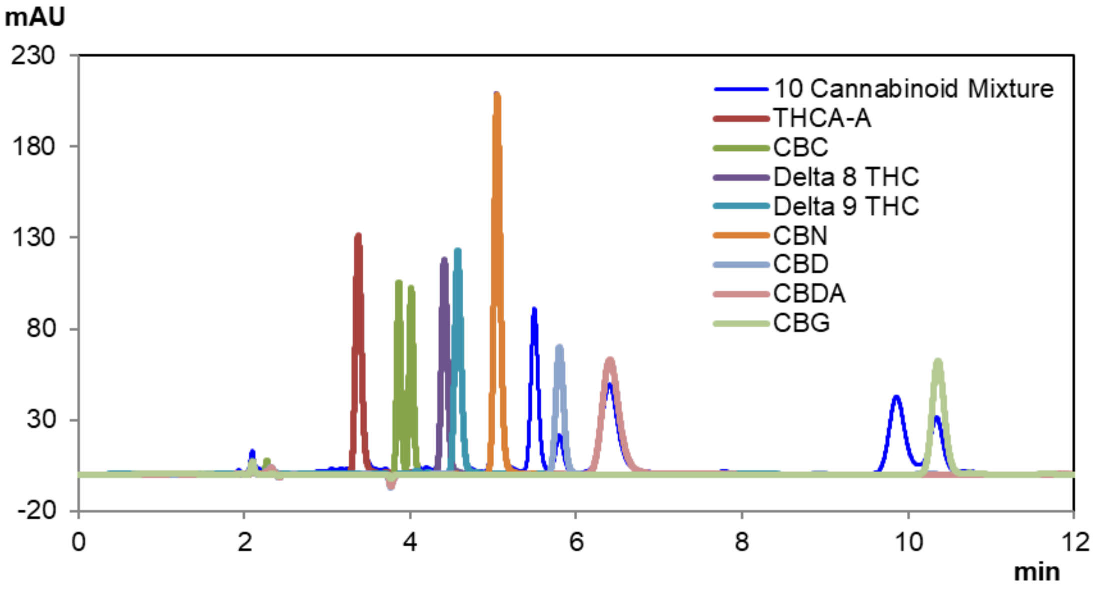


**Figure S4**: Peak Identification for the separation under normal phase conditions with Chiralpak IH-U

**Table S5**: Peak Identification for the separation under reversed phase conditions with Chiralpak IG-U – Analysis Conditions

| | Chiralpak IG-U |
|---|---|
| Column Dimension | 3.0 mm i.d. × 100 mm L |
| Mobile Phase | 45/55/0.1 = Water/Acetonitrile/Trifluoroacetic acid (v/v/v) |
| Flow Rate | 0.800 ml/min |
| Temperature | 25°C (controlled) |
| Detection | 220 nm UV |
| Sample | 10 cannabinoid mixture (1) 0.1 mg/ml in Water/ACN = 47.5/52.5 Individual Samples: 1.0 mg/ml in Water/ACN = 47.5/52.5 |
| Injection Volume | 10 cannabinoid mixture - 0.5 μl Individual Samples – 0.1 μl |

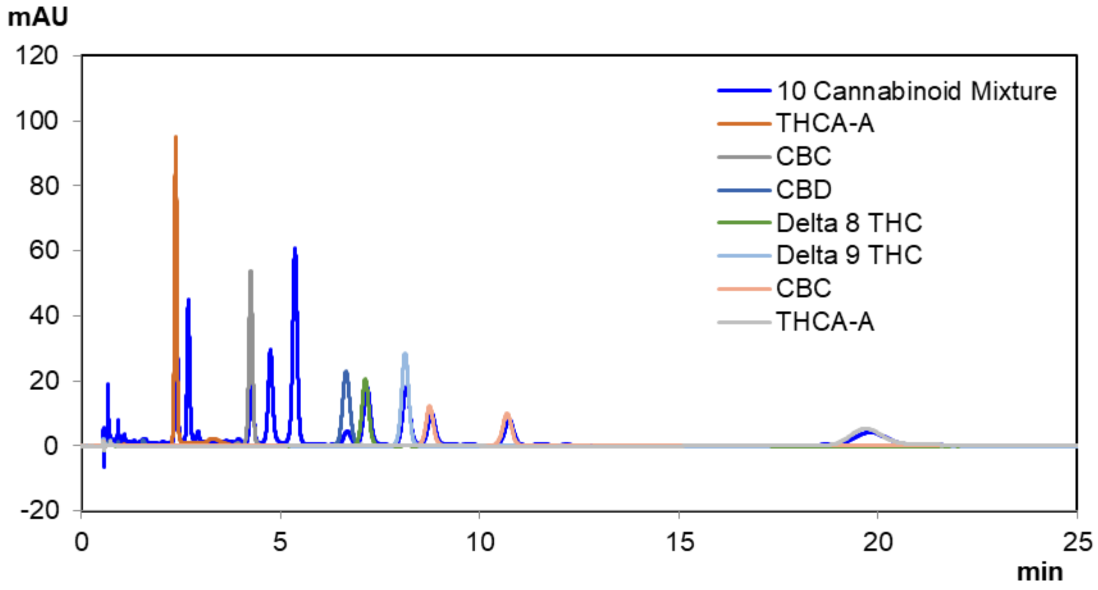


**Figure S5**: Peak Identification for the separation under reversed phase conditions with Chiralpak IG-U

**Table S6**: Peak Identification for the separation under reversed phase conditions with Chiralpak ID-U+IC-U – Analysis Conditions

| | **Chiralpak ID-U+IC-U** |
|---|---|
| Column Dimension | 3.0 mm i.d. × 100 mm L (2 columns coupled) |
| Mobile Phase | 42.5/57.5/0.1 = Water/Acetonitrile/Trifluoroacetic acid (v/v/v) |
| Flow Rate | 0.425 ml/min |
| Temperature | 25°C (controlled) |
| Detection | 220 nm UV |
| Sample | 10 cannabinoid mixture (1) 0.1 mg/ml in Water/ACN = 47.5/52.5<br>Individual Samples: 1.0 mg/ml in Water/ACN = 47.5/52.5 |
| Injection Volume | 10 cannabinoid mixture - 0.5 µl<br>Individual Samples – 0.1 µl |

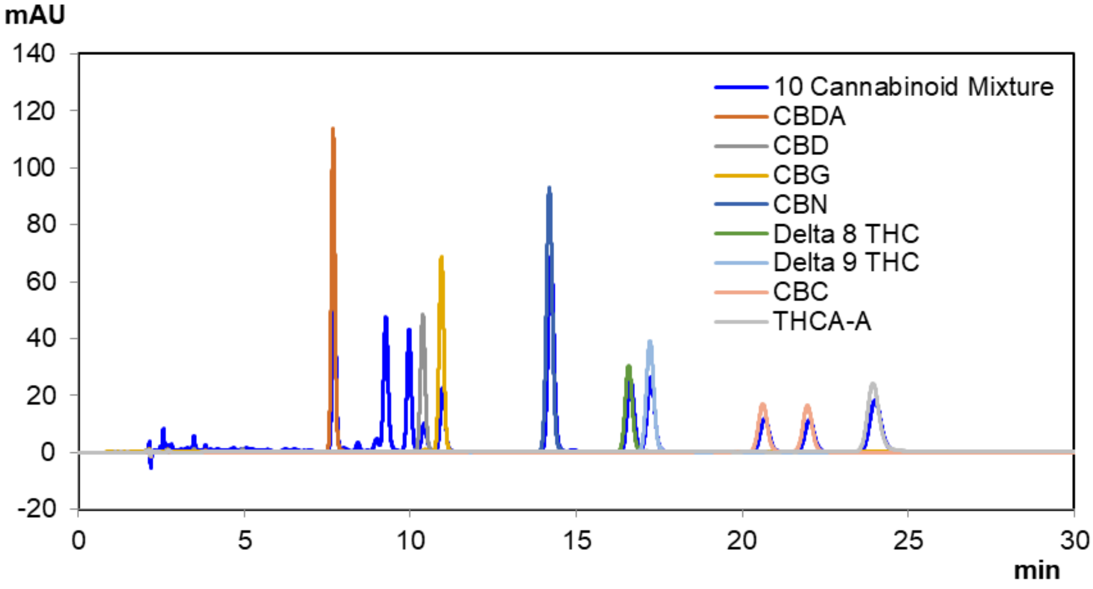


**Figure S6**: Peak Identification for the separation under reversed phase conditions with Chiralpak ID-U + IC-U

**Table S7**: Peak Identification for the separation under reversed phase conditions with Chiralpak ID-U – Analysis Conditions

| | Chiralpak ID-U |
|---|---|
| Column Dimension | 3.0 mm i.d. × 100 mm L |
| Mobile Phase | 55/45/0.1 = Water/Acetonitrile/Trifluoroacetic acid (v/v/v) |
| Flow Rate | 0.600 ml/min |
| Temperature | 25°C (controlled) |
| Detection | 220 nm UV |
| Sample | 10 cannabinoid mixture (1) 0.1 mg/ml in Water/ACN = 47.5/52.5<br>Individual Samples: 1.0 mg/ml in Water/ACN = 47.5/52.5 |
| Injection Volume | 10 cannabinoid mixture - 0.5 µl<br>Individual Samples – 0.1 µl |

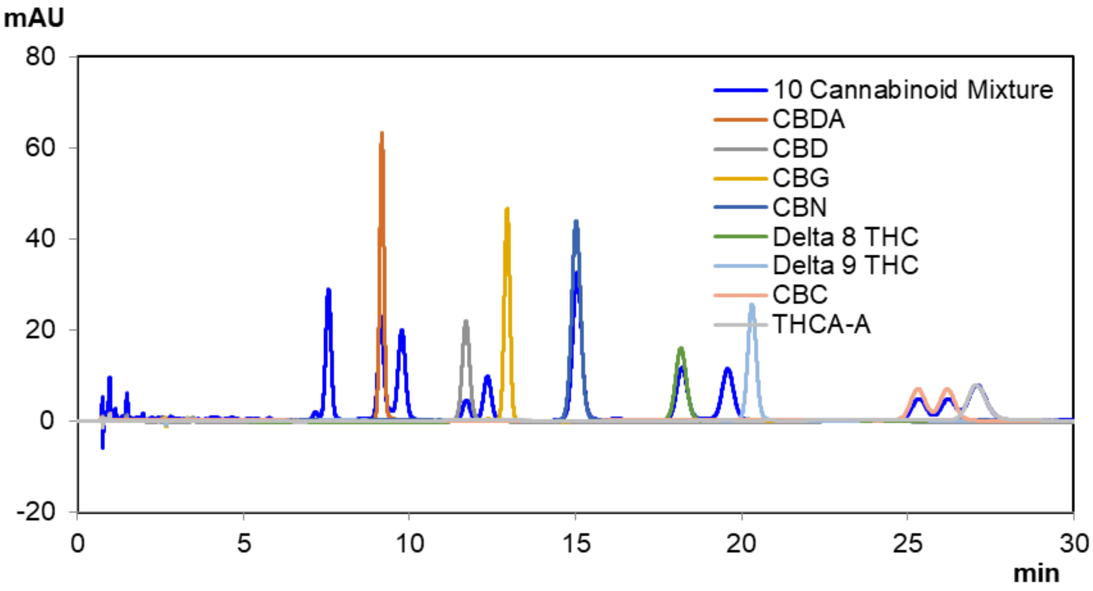


**Figure S7**: Peak Identification for the separation under reversed phase conditions with Chiralpak ID-U
